# Real-World Evidence in FDA Approvals for Labeling Expansion of Small Molecules and Biologics

**DOI:** 10.1007/s43441-025-00816-9

**Published:** 2025-06-04

**Authors:** Yung-Fang Deng, Cynthia J Girman, Mary E Ritchey

**Affiliations:** 1 CERobs Consulting, LLC, Asheville, NC USA; 2 Gillings School of Global Public Health, University of North Carolina at Chapel Hill, Chapel Hill, NC USA; 3 Center for Pharmacoepidemiology and Treatment Science, Rutgers University, New Brunswick, NJ USA

**Keywords:** Real-world evidence, Labeling expansion, Food and drug administration, Biologic license application, New drug application

## Abstract

**Introduction:**

Real-world evidence (RWE) can support the evaluation of safety and efficacy for medical products, but its extent of use in labeling expansion submissions remains unclear. This study aimed to characterize the RWE used in labeling expansion or likely used, as identified through literature search, for drugs and biologics.

**Methods:**

We identified RWE used in FDA-approved labeling expansion for drug and biologic supplemental applications from January 2022 to May 2024 (Drugs@FDA), using FDA prescribing information and review documents. We also searched ClinicalTrials.gov and PubMed to identify RWE not included in the FDA approval letter and labeling but could have been incorporated into submissions by sponsors. Characteristics of the RWE were extracted and summarized.

**Results:**

Among 218 labeling expansions granted, RWE was found in FDA documents for 3 approvals and elsewhere for 52 approvals. The proportion of approvals with RWE was 23.3%, 27.7%, and 23.7% in 2022, 2023, and 2024, respectively. RWE was most commonly found in submissions for oncology (43.6%), infection (9.1%), and dermatology (7.3%). Greater use of RWE was identified in submissions for drugs (69.1%) and to expand indications (78.2%). RWE came from 88 studies, with 48.9% addressing both safety and efficacy. Most of the RWE studies were retrospective (65.9%), employed a cohort study design (87.5%), and used electronic health records (EHR) data (75.0%).

**Conclusion:**

We observed limited RWE use in granted labeling expansion, and the reason is unclear due to incomplete FDA documentation of supplemental approvals—RWE may not have been submitted, may have been submitted but determined by FDA to be of limited use, may have contributed substantively to the supplemental approval, or some combination of these across submissions. Improving accessibility and transparency in RWE’s acceptability within review documents can enhance our understanding of the extent and quality of RWE used for labeling expansion.

**Supplementary Information:**

The online version contains supplementary material available at 10.1007/s43441-025-00816-9.

## Introduction

Real-world data (RWD), defined as data relating to patient health status and/or the delivery of healthcare, is routinely collected from various sources such as electronic health records (EHR), medical claims, and registries. Real-world evidence (RWE) pertains to clinical evidence derived from the analysis of RWD, focusing on the usage, potential benefits, or risks of medical products [1]. RWE has a long-standing history of supporting the post-market safety and, to a limited extent, the evaluation of the effectiveness of medical products. As complementary evidence to randomized clinical trials (RCTs), it typically offers better generalizability and efficiency in generating evidence [2]. Moreover, advancements in the analysis of RWD have expanded the potential for robust RWE to support regulatory decisions, particularly regarding effectiveness assessment [3]. In 2016, the enactment of the 21st Century Cures Act increased the attention given to RWE within the Food and Drug Administration’s (FDA) regulatory decision-making framework.

Recently, the use of RWE to support safety and efficacy evaluations in drug applications for regulatory decisions, both for original and supplemental (e.g., labeling expansion) submissions, has been increasing on a global scale [4–6]. Labeling expansions, a subset of labeling changes, refers specifically to decisions that include modifications expanding the applicable population by adding a new indication or expanding the existing indicated population for certain medical products. Labeling expansions usually involve safety and efficacy evaluations and can extend the applicability of an existing medical product in a more time- and cost-effective manner compared to the process involved in full development of a new drug [7, 8]. Regulators have issued guidance documents to raise awareness of their expectations and needs for reviewing such evidence [9–11]. Therefore, understanding RWE’s utilization, capabilities, and limitations could assist pharmaceutical stakeholders in better employing it in their endeavors to expand labeling.

A limited number of prior published studies have reviewed the FDA’s stance on using RWE for safety and efficacy assessment in new drug applications (NDAs) and biologics license applications (BLAs) [4, 5, 12–16]. However, no prior research has solely focused on the reliance on RWE in labeling expansion for drug products. To address this gap, a comprehensive review was conducted of supplemental applications for NDAs and BLAs submitted to the FDA, aiming to investigate the current status of RWE in supporting supplemental applications for labeling expansion.

## Methods

FDA publicly posts the regulatory team reviews for original NDAs and BLAs, which contain information on the clinical data used to support the submission. However, only the approval letter and labeling are publicly available for supplemental applications. Given the limited availability of public documents from the FDA, RWE studies which were likely to be available at the time of the supplemental application submission were identified through multiple resources, including FDA documents, ClinicalTrials.gov, and PubMed. We collected and summarized the characteristics of RWE used (as identified in FDA documents) or likely to be used (as identified through other resources) for labeling expansion applications.

### Eligibility Criteria

We reviewed approvals of supplemental NDAs (sNDAs) and BLAs (sBLAs) for labeling expansions from January 1, 2022 to May 31, 2024. Supplemental approvals with purposes other than adding an indication and expanding the indicated population (e.g., adding pediatric patients), as well as duplicated approvals, were excluded. No restrictions were placed based on therapeutic area, indication, indicated population, and designation.

### Search Strategy

A list of supplemental approvals was identified within the “supplemental approvals to NDAs and BLAs by month” section on Drugs@FDA, a database of drugs and biologics approved by the Center for Drug Evaluation and Research (CDER) [17]. To further identify labeling expansion approvals, we searched for publicly available documents such as approval letters and prescribing information first within Drugs@FDA and, when unavailable, in FDALabel, a prescribing information database of approved products [18]. These documents were reviewed for every sNDA and sBLA to determine the eligibility of labeling expansion.

After acquiring the list of labeling expansions, we searched for RWE that supported evidence of safety or effectiveness, regardless of whether it was sponsored by the applicants or received positive or negative comments from the FDA. First, we reviewed the documents available from FDA, such as prescribing information and review documents (integrated reviews, multidisciplinary reviews, clinical reviews, and statistical reviews) to identify RWE that have been included in these submissions. In addition, as FDA does not routinely and systematically mentioned RWE within the review documents for these supplemental applications and these details may not be easily accessible to the general public, we conducted searches on ClinicalTrials.gov (a database of worldwide clinical studies) [19] and PubMed to identify any relevant RWE that might have been included in these submissions but not mentioned in documents available from FDA. The searches were conducted for each sNDA and sBLA by using the corresponding product name, generic name, and National Clinical Trial (NCT) number (if applicable), as well as the purposes of labeling expansion (e.g., certain therapeutic areas or indicated populations added). Registered clinical research and peer-review articles published any time before the approval dates were reviewed.

### Data Extraction

The list of labeling expansions and RWE studies identified in the search were extracted with basic information on the product, labeling changes, and RWE characteristics using standardized data abstraction spreadsheets in Microsoft Excel (Supplemental Table 1). Specifically, study designs were classified into case series, cohort studies of patients receiving the treatment in the manner targeted by the label expansion (with or without comparison groups), external control studies, and natural history studies of disease (either with standard of care or without any intervention). Although we may not know the role of natural history studies in supporting safety or effectiveness in labeling expansion applications without information in the FDA documentation, we assume that such studies can be used to inform clinical trial design (e.g., justifying sample size, selecting control arms) and to support the interpretation of adverse events or outcomes observed in clinical trials by providing information on the natural course of a disease, including common complications or adverse events. Data sources were categorized as EHR, claims, registries, and patient-generated health data (PGHD), which includes adverse events, questionnaires, indices based on patient-reported information, and data collected by devices such as Fitbits, smartwatches, smartphones or tablets.

RWE studies were categorized into three subgroups based on their source and whether it was sponsored by the manufacturing company: (1) documents available from FDA, (2) non-FDA and non-manufacturer-sponsored (e.g., the manufacturers were not listed as sponsors or part of the authorship), (3) non-FDA and manufacturer-sponsored. Missing values were listed as unknown. Additionally, FDA’s Spectrum of Diseases/Conditions [20] was referenced to align classifications of therapeutic areas. This study collected publicly available reports that did not involve individual patient data and was not submitted for institutional review board approval.

Data collection and extraction were conducted by a single reviewer, which could introduce selection bias. To mitigate this, the reviewer was well-trained, and a second researcher was assigned to perform a selective examination after data extraction was completed.

### Statistics Analysis

Descriptive analyses were conducted to determine the proportion of labeling expansion approvals among all FDA supplemental approvals and the proportion of submissions that used or likely used RWE among all labeling expansion approvals. These analyses were further stratified by year, product type (NDA/BLA), therapeutic area, and the purpose of approval (adding an indication or expanding the indicated population) to investigate trends or differences among these categories.

Additionally, descriptive analyses were performed on RWE studies corresponding to labeling expansion approvals identified through searches in FDA documents, ClinicalTrials.gov, and PubMed. These analyses aimed to investigate the methods, data sources, and objectives of the RWE to comprehend how RWD has been employed to generate pertinent and credible evidence that might facilitate approved labeling expansion. Given the uncertainty regarding whether the information obtained from ClinicalTrials.gov and PubMed was included in the application packages for these approvals, subgroup analyses were conducted on the characteristics and utilization of RWE to identify differences between RWE from various sources and sponsorship. We reasonably assume that RWE sponsored by manufacturers, who were the applicants for the labeling expansions, would be more likely to be included in their submission packages. Furthermore, results of objectives, study designs, and data sources of RWE were stratified by year and therapeutic area. All analyses and data visualization were conducted in Microsoft Excel.

## Results

### Labeling Expansion Approvals with RWE

Among 3,326 supplemental approvals issued between January 1, 2022 and May 31, 2024, the review identified a total of 218 supplemental approvals aimed at expanding indications and populations. There were 141 (64.7%) NDAs and 77 (35.3%) BLAs; 142 (65.1%) were for *indication* expansion, and 76 (34.9%) were for *population* expansion (Table 1). The three therapeutic areas with the highest proportion of labeling expansion approvals were oncology (34.9%), infection (12.8%), and rheumatology (8.3%).

Among these 218 labeling expansion approvals, 55 (25.2%) were identified as having available RWE in FDA publicly available document and literature search (Table 1). Among the 55 approvals with RWE, 3 were identified in documents available from FDA and 52 were found from ClinicalTrials.gov and PubMed. NDAs (69.1%) and expansion of indications for both NDAs and BLAs (78.2%) were the majority of labeling expansion approvals with RWE. Furthermore, 6/23 (26.1%) of approvals for non-oncology orphan drugs were found to have available RWE, whereas 24/76 (31.6%) of oncology drug approvals were similarly identified.

**Table 1:** Characteristics of *FDA supplemental approvals for labeling expansion*, 2022–2024 (*N* = 218)

Characteristic	Approvals with RWE	Total approvals for labeling expansion
	(*n* = 55)	(*n* = 218)
	*n* (%)	*n* (%)
Purpose of approval		
Add an indication	43 (78.18)	142 (65.14)
Expand the intended population	12 (21.82)	76 (34.86)
Product type		
NDA	38 (69.09)	141 (64.68)
BLA	17 (30.91)	77 (35.32)
Approved year		
2022	20 (36.36)	86 (39.45)
2023	26 (47.27)	94 (43.12)
2024	9 (16.36)	38 (17.43)
Designation ^a^		
Orphan drug	17 (30.91)	70 (32.11)
Pediatric	7 (12.73)	33 (15.14)
Genetic	7 (12.73)	25 (11.47)
Therapeutic area		
Allergy	1 (1.82)	1 (0.46)
Analgesia	0 (0.00)	3 (1.38)
Cardiovascular Disease	3 (5.45)	5 (2.29)
Dermatology	4 (7.27)	16 (7.34)
Endocrinology/Metabolism	3 (5.45)	17 (7.80)
Gastroenterology	2 (3.64)	4 (1.83)
Hematology	2 (3.64)	7 (3.21)
Immunomodulators	0 (0.00)	2 (0.92)
Infection	5 (9.09)	28 (12.84)
Medical Imaging	3 (5.45)	6 (2.75)
Neurology	0 (0.00)	6 (2.75)
Oncology	24 (43.64)	76 (34.86)
Ophthalmology	1 (1.82)	8 (3.67)
Other	3 (5.45)	12 (5.50)
Psychiatry	1 (1.82)	6 (2.75)
Pulmonary	1 (1.82)	3 (1.38)
Rheumatology	2 (3.64)	18 (8.26)

Abbreviation: RWE, Real-world evidence; FDA, U.S. Food and Drug Administration; NDA, new drug application; BLA, biologics license application. ^a^ Each designation was not mutually exclusive, one supplemental approval could have more than one designation

**Fig. 1:**
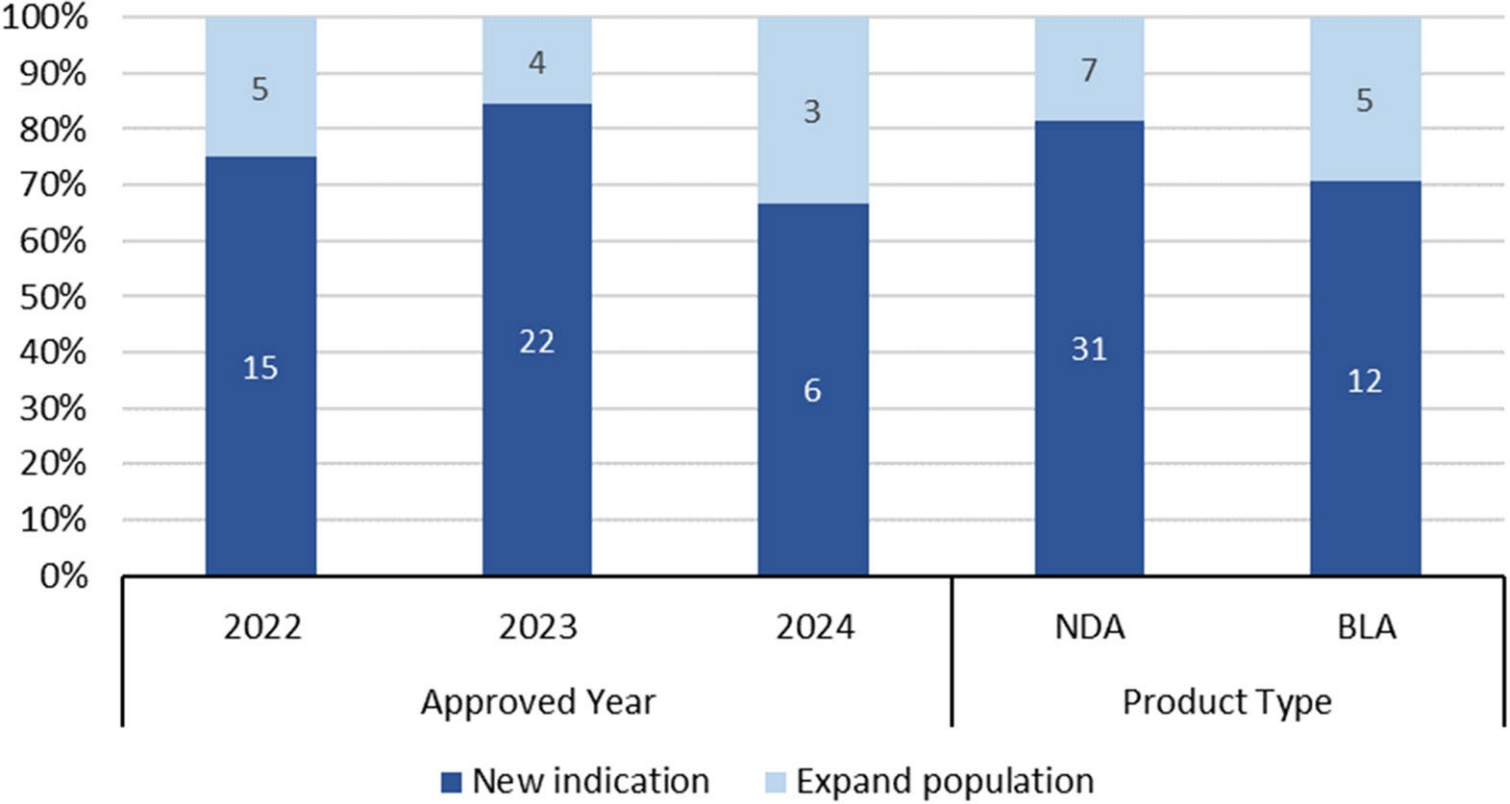
Purpose of Approvals with RWE by Year and Product Type. Abbreviation: NDA: new drug application; BLA: biologics license application

The proportion of approvals with RWE, stratified by years, suggests a potentially increasing role of RWE in evaluating safety and efficacy of medical products, with 26/94 (27.7%) in 2023 and 9/38 (23.7%) in 2024, compared to 20/86 (23.3%) in 2022 (Supplemental Table 2). Meanwhile, from 2022 to 2024, RWE was more commonly available as evidence for indication expansion, and variable across years (Fig. 1). NDAs showed a higher proportion of approvals with available RWE for expanding indications compared to BLAs (31/38, 81.6% versus 12/17, 70.6%, respectively).

**Fig. 2:**
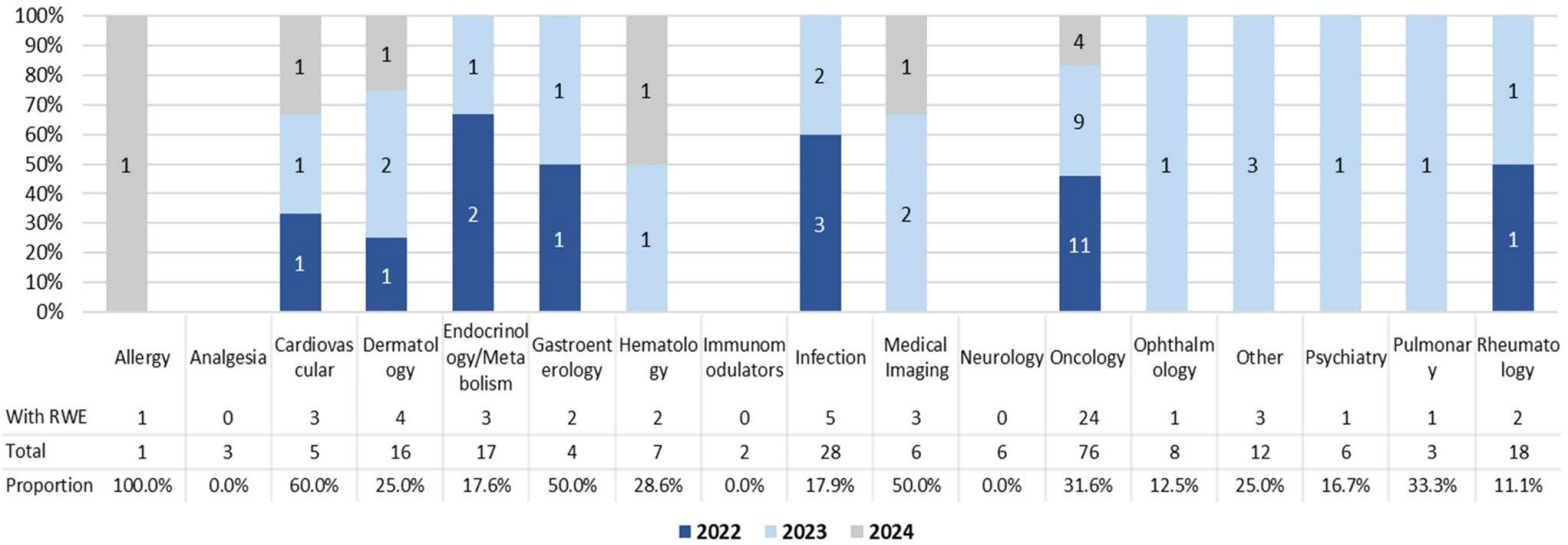
Proportion of Approvals with RWE by Therapeutic Area

Oncology was the therapeutic area most frequently reported to have included RWE among all labeling expansion approvals (43.6%), followed by infection and dermatology (9.1% and 7.3%, respectively) (Table 1). Among the approvals for each therapeutic area, allergy (100.0%), cardiovascular disease (60.0%), gastroenterology (50.0%), and medical imaging (50.0%) had the highest proportions of approvals that included RWE (Fig. 2). For therapeutic areas with more than 15 approvals, the proportion of approvals with available RWE ranged from 11.1 to 31.6%, with oncology having the highest proportion. Availability of RWE in approvals across therapeutic areas was seen most frequently in 2023 (10 out of 16 therapeutic areas).

### Characteristics of RWE

Through publicly available FDA documents and literature searches, 88 RWE studies were available for the 55 labeling expansion approvals (Table 2). Only 4 RWE studies were identified in the documents available from the FDA. Among all identified RWE studies, 13 (14.8%) primarily focused on safety only, 32 (36.3%) on efficacy only, and 43 (48.9%) addressed both safety and efficacy. Across all 3 years, RWE was often available to support both safety and efficacy objectives (50.0% in 2022, 46.7% in 2023, and 54.6% in 2024) (Table 3).

**Table 2:** Characteristics of RWE for FDA supplemental approvals for labeling expansion, 2022–2024 (*N* = 88)

Characteristic	RWE from review documents or prescribing information	RWE from ClinicalTrials.gov or PubMed that was not sponsored by manufacturing company	RWE from ClinicalTrials.gov or PubMed that sponsored by manufacturing company	Total RWE
	(*n* = 4)	(*n* = 57)	(*n* = 27)	(*n* = 88)
	*n* (%)	*n* (%)	*n* (%)	*n* (%)
Objective of RWE				
Safety	3 (75.00)	4 (7.02)	6 (22.22)	13 (14.77)
Efficacy	0 (0.00)	21 (36.84)	11 (40.74)	32 (36.36)
Both safety and efficacy	1 (25.00)	32 (56.14)	10 (37.04)	43 (48.86)
Study design				
Prospective	2 (50.00)	15 (26.32)	12 (44.44)	29 (32.95)
Retrospective	2 (50.00)	41 (71.93)	15 (55.56)	58 (65.91)
Hybrid	0 (0.00)	1 (1.75)	0 (0.00)	1 (1.14)
Case series	0 (0.00)	6 (10.53)	1 (3.70)	7 (7.95)
Cohort study	3 (75.00)	51 (89.47)	23 (85.19)	77 (87.50)
External control	0 (0.00)	0 (0.00)	2 (7.41)	2 (2.27)
Natural history study	1 (25.00)	0 (0.00)	1 (3.70)	2 (2.27)
Data sources				
EHR only	1 (25.00)	46 (80.70)	19 (70.37)	66 (75.00)
Claims only	0 (0.00)	2 (3.51)	0 (0.00)	2 (2.27)
Registry only	0 (0.00)	0 (0.00)	4 (14.81)	4 (4.55)
PGHD only	0 (0.00)	1 (1.75)	2 (7.41)	3 (3.41)
EHR and PGHD	0 (0.00)	5 (8.77)	0 (0.00)	5 (5.68)
EHR and Registry	1 (25.00)	1 (1.75)	1 (3.70)	3 (3.41)
EHR and Claims	0 (0.00)	0 (0.00)	1 (3.70)	1 (1.14)
Claims and Registry	0 (0.00)	1 (1.75)	0 (0.00)	1 (1.14)
Not reported	2 (50.00)	1 (1.75)	0 (0.00)	3 (3.41)
Approved year				
2022	1 (25.00)	16 (28.07)	15 (55.56)	32 (36.36)
2023	2 (50.00)	34 (59.65)	9 (33.33)	45 (51.14)
2024	1 (25.00)	7 (12.28)	3 (11.11)	11 (12.50)
Sources of RWE				
Prescribing information	1 (25.00)	0 (0.00)	0 (0.00)	1 (1.14)
Review documents	3 (75.00)	0 (0.00)	0 (0.00)	3 (3.41)
ClinicalTrials.gov or PubMed	0 (0.00)	57 (100.00)	27 (100.00)	84 (95.45)

Abbreviation: RWE, Real-world evidence; FDA, U.S. Food and Drug Administration; EHR, electronic health record; PGHD, Patient-generated health data

**Table 3:** Purpose of RWE for FDA supplemental approvals for labeling expansion, 2022–2024 (*N* = 88)

Characteristic	2022	2023	2024
	(*n* = 32)	(*n* = 45)	(*n* = 11)
	*n* (%)	*n* (%)	*n* (%)
Objective of RWE			
Safety	4 (12.50)	9 (20.00)	0 (0.00)
Efficacy	12 (37.50)	15 (33.33)	5 (45.45)
Both safety and efficacy	16 (50.00)	21 (46.67)	6 (54.55)

Abbreviation: RWE, Real-world evidence; FDA, U.S. Food and Drug Administration

Among the 88 RWE studies, EHR (75%) was the most frequently used data sources, followed by the combinations of EHR and PGHD (5.7%) and registry data (4.6%) (Table 2). EHR was the most frequently used data sources across all years, with 31/32 (96.7%) in 2022, 26/45 (57.8%) in 2023, and 9/11 (81.8%) in 2024 (Supplemental Fig. 1). Additionally, EHR was the most frequently employed data source across most therapeutic areas, except for allergy, psychiatric, pulmonary, and rheumatology areas. Specifically for these therapeutic areas, the most commonly used data sources for RWE were: the combination of EHR and PGHD in the allergy therapeutic area, PGHD in the psychiatric area, registry data in the pulmonary area, and claims data in rheumatology area.

**Fig. 3:**
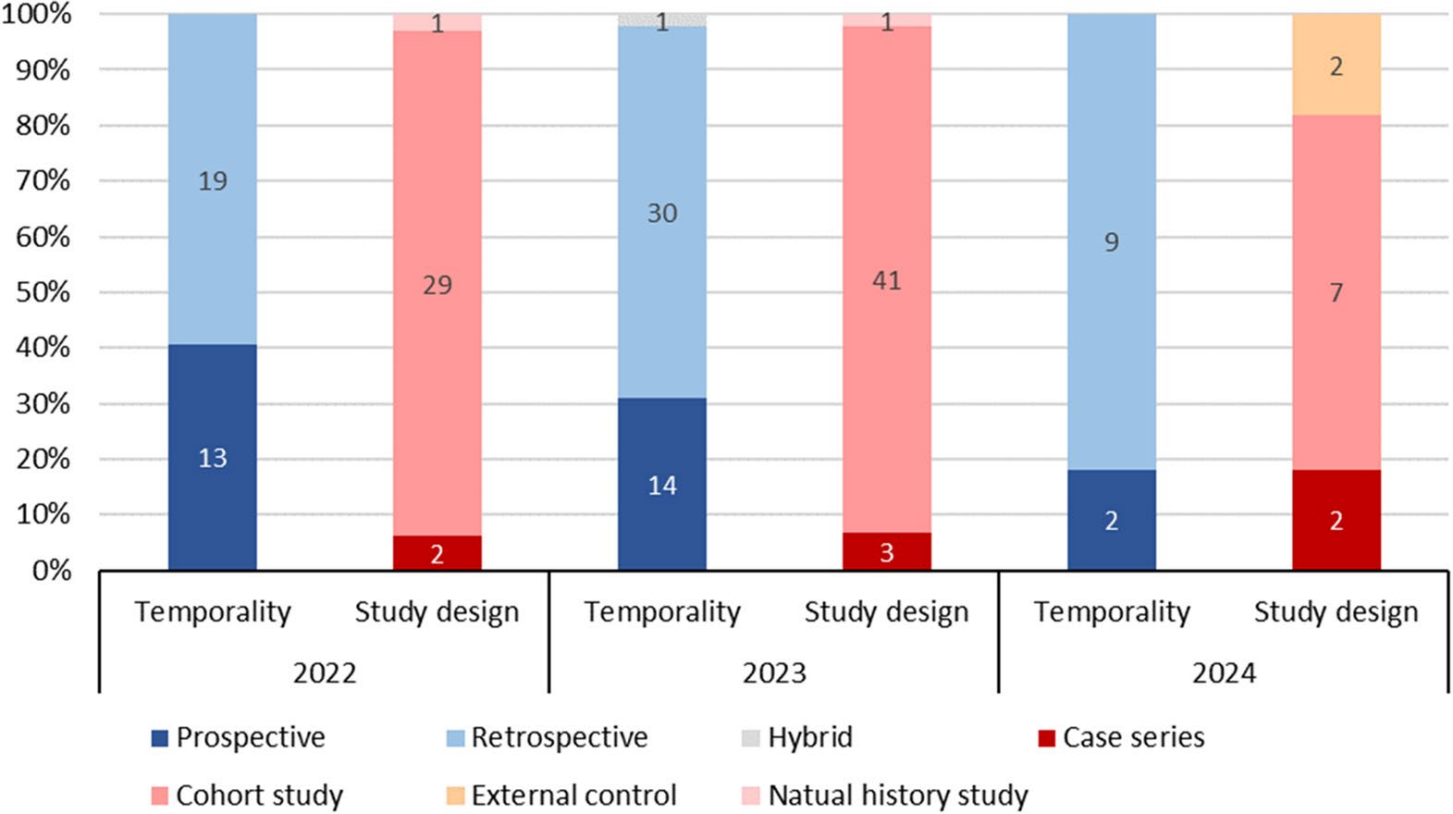
RWE Studies by Study Design and Year

**Fig. 4:**
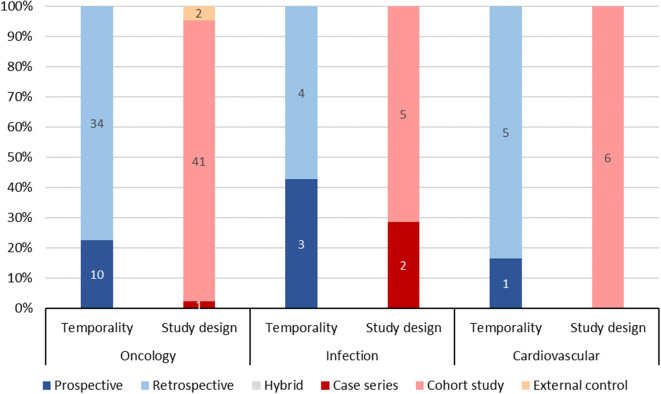
RWE Studies by Study Design and Therapeutic Area

The most commonly used study design for the RWE studies was cohort study (87.5%), followed by case series (8.0%), external control (2.3%), and natural history study (2.3%) (Table 2). Cohort studies remained the most frequently used study design across all years, with 29/32 (90.6%) in 2022, 41/45 (91.1%) in 2023, and 7/11 (63.6%) in 2024 (Fig. 3). Furthermore, cohort studies were the most commonly used study design across the three therapeutic areas with the highest number of RWE studies, with 41/44 (93.2%) in oncology, 5/7 (71.4%) in infection, and 6/6 (100.0%) in cardiovascular disease (Fig. 4).

The most frequently used temporality for the RWE studies was retrospective (65.9%), followed by prospective (33.0%) and hybrid (1.1%) (Table 2). Retrospective studies remained the most commonly used across all years. Moreover, the use of retrospective studies has been increasing, with 19/32 (59.4%) in 2022, 30/45 (66.7%) in 2023, and 9/11 (81.8%) in 2024 (Fig. 3). Retrospective studies were also the most commonly used study temporality across the three therapeutic areas with the highest number of RWE studies, with 34/44 (77.3%) in oncology, 4/7(57.1%) in infection, and 5/6 (83.3%) in cardiovascular disease (Fig. 4).

Lastly, we investigated the methods employed to address missing data and bias in each RWE study. Among all RWE, 36.3% reported methods for handling missing data, 39.8% reported their approaches for bias assessment, and 21.6% reported both.

### RWE from Different Sources

Only a limited number of publicly available documents from FDA featured the role of RWE. Among the 52 labeling expansion approvals with RWE found in ClinicalTrials.gov and PubMed, 19 (36.5%) had at least one RWE source identified as manufacturer-sponsored. Among the 88 RWE studies, 3 RWE studies derived from review documents and 1 from prescribing information from FDA (Table 2). Of these, only two RWE studies for the same sNDA (REVATIO) were discussed in the FDA review documents [21], with one indicated by FDA reviewers as evidence of adverse events [22] and the other noted as unpublished results.

Furthermore, 27 of RWE studies were non-FDA and manufacturer-sponsored, while 55 were non-FDA and non-manufacturer-sponsored. Across the three subgroups of RWE, similar features were observed in terms of the predominantly employed study designs and data sources. RWE sourced from documents available from FDA were more likely to be used solely for safety objectives (75.0%). In contrast, non-FDA and manufacturer-sponsored RWE was more frequently used for efficacy objectives only (40.7%), while non-FDA and non-manufacturer-sponsored RWE was more often used for both efficacy and safety concurrently (56.1%).

## Discussion

According to our study, 25.2% of approved supplemental submissions for labeling expansion likely included RWE (with only 1.4% documented by the FDA). Although no prior study has explored the extent of RWE use in labeling expansion submissions, a few studies have examined RWE use among approved submissions for novel medical products (i.e., original approvals). For instance, Purpura et al. reported that 64.7% of original FDA approvals of NDAs or BLAs incorporated RWE to provide evidence of product safety or efficacy [16]. Lau et al. reported that 68.4% of original approvals for non-oncology orphan drugs and 75.9% for oncology products included RWE in submission packages [4]. Bloomfield-Clagett et al. reported that 10.0% of original approvals for neuroscience-related submissions included RWE [13].

Compared to these studies, the proportion of RWE use in our study for the same therapeutic areas or designations were substantially lower. This may indicate a lower proportion of RWE use in labeling expansion approvals (or supplemental approvals in general) compared to that in original approvals. In a study of FDA approvals in 1954–2020 that used RWE to support product efficacy, more RWE-included approvals were for original approvals (*n* = 30) compared to supplemental approvals (*n* = 4) [15]. However, it is also possible that we underestimated the use of RWE in labeling expansion approvals, as the FDA does not routinely release review documents for supplemental approvals. To capture approvals that used RWE but were not noted in FDA documents, we leveraged other data sources (e.g., ClinicalTrials.gov, PubMed) but might have still missed some RWE-assisted approvals. In addition, we could have identified RWE studies that were conducted but not considered by FDA in their review of the sNDA or sBLA.

Our study observed the use of RWE across various therapeutic areas. Oncology comprised the largest portion of all supplemental approvals that included RWE, followed by infection and dermatology. Although no study has examined the distribution of therapeutic areas exclusively among labeling expansion approvals that included RWE, research on FDA original and supplemental approvals between 1954 and 2020 indicated that the therapeutic areas with the highest number of RWE-included approvals were oncology, hematology, and neurology [15]. Research on FDA original approvals for NDAs and BLAs in 2019–2021 found that submissions for oncology, infectious diseases, and neurological diseases most commonly included RWE [16]. Additionally, a research study, which tracked published RWE studies in 2017, found that oncology, infectious diseases, and cardiovascular diseases had the highest volume of publications related to drug evaluations [23]. Our study is generally consistent with these studies in demonstrating oncology as the predominant therapeutic area where RWE is used or possibly used in submissions. However, the therapeutic areas that were reported as having a larger proportion of approvals including RWE varied across studies. One reason that may explain the discrepancies is variations in the distribution of therapeutic areas among approvals across different study periods [24]. For instance, in response to the emergence of COVID-19, the FDA established the Coronavirus Treatment Acceleration Program (CTAP) and formed the Accelerating COVID-19 Therapeutic Interventions and Vaccines (ACTIV) partnership between the private and public sectors in 2020 [25, 26]. Specifically, CTAP was designed to provide a rapid review of potential treatments (other than vaccines) and make them available to patients. Improved efficiency in processing approvals for treatments, such as antiviral agents, may have led to a different distribution of approvals across therapeutic areas during that period [27]. Additionally, the distribution of therapeutic areas among approvals differed between original approvals and supplemental approvals, with more than half of the supplemental approvals being for oncology treatments [28, 29]. Differences in definitions or reference frameworks for classifying therapeutic areas may also partially account for the discrepancies. For instance, hematologic tumors might be categorized as hematology in some studies, whereas we classified them under oncology.

Among therapeutic areas with more than 15 supplemental approvals, the proportion of approvals with available RWE ranged from 11.1 to 31.6%, with oncology having the highest proportion. These results suggest that oncology not only had the most submissions for labeling expansion but also utilized RWE more extensively compared to other therapeutic areas.

Historically, RWE has been instrumental in supporting the monitoring of post-market safety and generating clinical evidence of drug efficacy, especially when RCTs were impractical or ethically challenging [12]. The FDA also reported that in 2023 submissions to the CDER, RWE was used to support either effectiveness or safety [30]. Our study found that of the 48.9% of labeling expansions in supplemental applications using or possibly using RWE to support both safety and efficacy, 36.3% were focused exclusively on efficacy, and only 14.8% were focused solely on safety.

Few studies have examined the objectives of RWE in medical product submissions. Research on FDA original approvals for NDAs and BLAs in 2019–2021 indicated that, although RWE was generally more commonly used to support safety during this period, the proportion of RWE studies supporting both safety and effectiveness increased, surpassing the proportion used for safety alone by 2021 [16]. Although these results may suggest that RWE was increasingly used to support both safety and effectiveness of medical products, differences in study periods and the focus on original versus supplemental applications prevent us from drawing definitive conclusions.

Understanding the data sources and study designs of RWE is crucial for its future application. Our results indicated that the majority of RWE relied on EHR data. Previous literature shows mixed findings regarding the data sources used in RWE studies for evaluating medical products. For instance, Vaghela et al. identified registry data as the most common source of RWE for NDAs and BLAs related to rare diseases [5]. Another research paper on published RWE studies for drug products found that existing data sources, such as medical records or administrative/pharmacy data, were the most commonly used [23]. The discrepancy is less likely due to uncertainty about whether the RWE studies were actually included in the submissions, which is a limitation of our study design. Our subgroup analyses revealed that RWE studies from FDA documents, as well as those from non-FDA and non-manufacturer-sponsored sources and non-FDA and manufacturer-sponsored sources, shared similar features in terms of commonly used data sources. However, the limited number of RWE studies from FDA documents may not fully represent the characteristics of the submissions and restricts our ability to address concerns about data quality—concerns highlighted in previous studies [14–16]—that could influence FDA regulatory decisions.

Our results also showed that RWE were predominantly retrospective (65.9%) and mainly cohort studies (87.5%). Although there were few studies including information on RWE study design, our findings align with those that indicated RWE are primarily retrospective [5, 23].

Like previous research [6, 16], our study agrees that RWE is increasingly being incorporated into the regulatory decision-making process. Although our study covers a relatively short time period of FDA supplemental approvals, it includes data from the year following 2022, providing the most up-to-date information on the role and utilization of RWE. To our knowledge, this study is the first to focus on the role of RWE in supplemental approvals for labeling expansion of indications and intended populations. Additionally, our study incorporated all therapeutic areas, allowing us to provide a broader perspective on the potential power and roles of RWE.

There are some limitations for our study. First, the RWE we identified may not fully represent the RWE included in submission packages to the FDA. This is primarily due to the limited publicly available review documents released by FDA, which led us to extend our search to ClinicalTrials.gov and PubMed. To ensure clarity, we carefully interpreted our results. Additionally, we conducted subgroup analyses based on the origin of the RWE. Under the assumption that RWE studies sponsored by manufacturing companies are more likely to be included in submission packages compared to non-sponsored studies, our finding indicated that the characteristics of study designs and data sources were similar across groups, whereas the objectives of RWE differed in their distribution. However, the small sample size of RWE identified on FDA websites limited our ability to have meaningful interpretation of RWE from other sources. Therefore, our findings are not intended to be interpreted as evidence of trends in RWE used in FDA labeling expansion applications; rather, they contribute to the understanding that EHR, retrospective studies, and cohort designs were the most commonly used data source and study design for RWE which would have been available to FDA reviewers (and potentially in the submission) at the time of the labeling expansion applications. Consequently, we cannot definitively determine the role of RWE intended by the sponsor or in the FDA reviewer’s consideration in labeling expansion based on our study. Nonetheless, we acknowledge that there are likely more RWE studies than those disclosed by the FDA, which could provide evidence on the safety and efficacy of medical products.

Another limitation of our study is the short observation period and the fact that 2024 was not a complete year. This restricts our ability to analyze annual trends and makes direct comparisons with results from other years difficult.

## Conclusion

In conclusion, our comprehensive review described the role and characteristics of RWE in FDA supplemental approvals for labeling expansion. We found that 25.2% of labeling expansion approvals included or likely included RWE. Of these, RWE was more likely to be used for supplemental NDA (sNDA) submissions and for expanding indications of products. Labeling expansion approvals with available RWE were predominantly for oncology products. Most RWE studies addressed both safety and efficacy, employed retrospective designs, and included a substantial proportion of cohort studies. Additionally, EHRs were the primary data source for RWE.

However, more information from FDA is needed to explore how RWE is applied in labeling expansions, particularly in the context of supplemental submission packages. The FDA has published guidelines outlining the use of RWE to bolster regulatory decisions for medical devices, NDAs, and BLAs, aiming to accelerate the development of medical products and deliver new innovations and advancements to patients [10, 31, 32]. Additionally, the FDA’s RWE Program has established a framework for assessing the feasibility of RWE in evaluating product effectiveness, which includes three key approaches: evaluating the suitability of Real-World Data (RWD) to address the scientific research question(s), assessing the scientific rigor of the trial or study design that generates RWE, and ensuring compliance with FDA regulatory standards [1]. A guideline published by the FDA in 2022 further encouraged sponsors and applicants to indicate the use of RWD/RWE in their submission cover letters [33]. Despite these guidelines and initiatives, there are limited examples and resources available to fully understand how this framework and these guidelines are implemented in practice for regulatory decisions on labeling expansion. Therefore, we anticipate obtaining further insights and resources from the FDA for a more comprehensive evaluation of their standards and concerns, such as data quality and methodological issues, when making regulatory decisions based on applications that include RWE.

## Electronic Supplementary Material

Below is the link to the electronic supplementary material.

Supplementary Material 1

## Data Availability

Data that suppport the findings of this study were obtained from Drugs@FDA, FDALable, ClinicalTrials.gov, and PubMed, which are publicly available databases.

## References

[1] FDA. Framework for FDA’s Real-World Evidence Program [Internet]. 2018 [cited 2024 May 21]. Available from: https://www.fda.gov/media/120060/download?attachment

[2] Mahendraratnam N, Mercon K, Eckert J, Romine M, Kroetsch A, Frank K et al. Adding Real-World Evidence to a Totality of Evidence Approach for Evaluating Marketed Product Effectiveness [Internet]. 2019 [cited 2024 May 21]. Available from: https://healthpolicy.duke.edu/sites/default/files/2020-08/Totality%20of%20Evidence%20Approach.pdf

[3] FDA. Real-World Evidence [Internet]. FDA. FDA. 2023 [cited 2024 Aug 14]. Available from: https://www.fda.gov/science-research/science-and-research-special-topics/real-world-evidence

[4] Lau C, Jamali F, Loebenberg R. Health Canada usage of real world evidence (RWE) in regulatory decision making compared with FDA/EMA usage based on publicly available information. J Pharm Pharm Sci Publ Can Soc Pharm Sci Soc Can Sci Pharm. 2022;25:227–36. 10.18433/jpps32715 35760071

[5] Vaghela S, Tanni KA, Banerjee G, Sikirica V. A systematic review of real-world evidence (RWE) supportive of new drug and biologic license application approvals in rare diseases. Orphanet J Rare Dis. 2024;19(1):117. 38475874 10.1186/s13023-024-03111-2 PMC10936002

[6] Mofid S, Bolislis WR, Kühler TC. Real-World data in the postapproval setting as applied by the EMA and the US FDA. Clin Ther. 2022;44(2):306–22. 35074209 10.1016/j.clinthera.2021.12.010

[7] Schlander M, Hernandez-Villafuerte K, Cheng CY, Mestre-Ferrandiz J, Baumann M. How much does it cost to research and develop a new drug? A systematic review and assessment. PharmacoEconomics. 2021;39(11):1243–69. 34368939 10.1007/s40273-021-01065-y PMC8516790

[8] Van Norman GA. Drugs, devices, and the FDA: part 1: an overview of approval processes for drugs. JACC Basic Transl Sci. 2016;1(3):170–9. 30167510 10.1016/j.jacbts.2016.03.002 PMC6113160

[9] FDA. Real-World Data: Assessing Electronic Health Records and Medical Claims Data To Support Regulatory Decision-Making for Drug and Biological Products [Internet]. FDA. 2024 [cited 2024 Sep 28]. Available from: https://www.fda.gov/regulatory-information/search-fda-guidance-documents/real-world-data-assessing-electronic-health-records-and-medical-claims-data-support-regulatory 10.1002/pds.5444 PMC9320939 35471704

[10] FDA. Use of Real-World Evidence to Support Regulatory Decision-Making for Medical Devices [Internet]. 2017 [cited 2024 May 21]. Available from: https://www.fda.gov/media/99447/download?attachment

[11] EMA. Data quality framework for medicines regulation [Internet]. 2022 [cited 2024 Sep 28]. Available from: https://www.ema.europa.eu/en/about-us/how-we-work/big-data/data-quality-framework-medicines-regulation

[12] Agrawal S, Arora S, Amiri-Kordestani L, de Claro RA, Fashoyin-Aje L, Gormley N, et al. Use of Single-Arm trials for US food and drug administration drug approval in oncology, 2002–2021. JAMA Oncol. 2023;9(2):266–72. 36580315 10.1001/jamaoncol.2022.5985

[13] Bloomfield-Clagett B, Rahman M, Smith K, Concato J. Use of Real-World evidence in Neuroscience-Related new drug and biologics license applications for novel therapeutics. Clin Pharmacol Ther. 2023;114(5):1002–5. 37548904 10.1002/cpt.3018

[14] Feinberg BA, Gajra A, Zettler ME, Phillips TD, Phillips EG, Kish JK. Use of Real-World evidence to support FDA approval of oncology drugs. Value Health J Int Soc Pharmacoeconomics Outcomes Res. 2020;23(10):1358–65. 10.1016/j.jval.2020.06.006 33032780

[15] Mahendraratnam N, Mercon K, Gill M, Benzing L, McClellan MB. Understanding use of Real-World data and Real-World evidence to support regulatory decisions on medical product effectiveness. Clin Pharmacol Ther. 2022;111(1):150–4. 33891318 10.1002/cpt.2272

[16] Purpura CA, Garry EM, Honig N, Case A, Rassen JA. The role of Real-World evidence in FDA‐Approved new drug and biologics license applications. Clin Pharmacol Ther. 2022;111(1):135–44. 34726771 10.1002/cpt.2474 PMC9299054

[17] FDA, Drugs@FDA. FDA-Approved Drugs [Internet]. [cited 2024 May 22]. Available from: https://www.accessdata.fda.gov/scripts/cder/daf/index.cfm?event=reportsSearch.process

[18] FDA. FDALabel [Internet]. [cited 2024 May 22]. Available from: https://nctr-crs.fda.gov/fdalabel/ui/search

[19] NCBI. ClinicalTrials.gov [Internet]. [cited 2024 May 22]. Available from: https://clinicaltrials.gov/

[20] FDA. Spectrum of Diseases by Therapeutic Area found in Written Requests as of December 31, 2019. 2020 Jan 28 [cited 2024 May 22]; Available from: https://www.fda.gov/drugs/development-resources/spectrum-diseasesconditions

[21] VIATRIS. REVATIO (SILDENAFIL CITRATE). [package insert] [Internet]. U.S. Food and Drug Administration website. 2023 [cited 2025 Mar 22]. Available from: https://www.accessdata.fda.gov/scripts/cder/daf/index.cfm?event=overview.process&ApplNo=021845

[22] Pfizer’s Upjohn has merged with Mylan to form Viatris Inc. Special Investigation For Long-Term Use Of Sildenafil (Regulatory Post Marketing Commitment Plan) [Internet]. clinicaltrials.gov; 2021 Feb [cited 2025 Mar 22]. Report No.: NCT00666198. Available from: https://clinicaltrials.gov/study/NCT00666198

[23] Ritchey ME, Buck P, Castro C, Fernandez M, Hollis K, Mordin M. Real-World Evidence for Drugs and Devices: 2017 Literature Reviewed. In 2018.

[24] Brown DG, Wobst HJ. A decade of FDA-Approved drugs (2010–2019): trends and future directions. J Med Chem. 2021;64(5):2312–38. 33617254 10.1021/acs.jmedchem.0c01516

[25] FDA. Coronavirus Treatment Acceleration Program (CTAP) [Internet]. Coronavirus (COVID-19) drugs. FDA. 2023 [cited 2024 Aug 17]. Available from: https://www.fda.gov/drugs/coronavirus-covid-19-drugs/coronavirus-treatment-acceleration-program-ctap

[26] NIH. NIH to launch public-private partnership to speed COVID-19 vaccine and treatment options [Internet]. News Releases 2020 April 17. 2020 [cited 2024 Aug 17]. Available from: https://www.nih.gov/news-events/news-releases/nih-launch-public-private-partnership-speed-covid-19-vaccine-treatment-options

[27] Cassidy C, Dever D, Stanbery L, Edelman G, Dworkin L, Nemunaitis J. FDA efficiency for approval process of COVID-19 therapeutics. Infect Agent Cancer. 2020;15:73. 33292374 10.1186/s13027-020-00338-z PMC7705854

[28] IQVIA. Global Oncology Trends 2018: Innovation, Expansion and Disruption [Internet]. 2018 [cited 2024 Aug 17]. Available from: https://www.iqvia.com/insights/the-iqvia-institute/reports-and-publications/reports/global-oncology-trends-2018

[29] Harris E. Few supplemental indications for drugs have added therapeutic value. JAMA. 2023;330(5):401. 37436780 10.1001/jama.2023.12539

[30] FDA. Real-World Evidence Submissions to the Center for Drug Evaluation and Research. FDA [Internet]. 2024 Jun 26 [cited 2024 Aug 12]; Available from: https://www.fda.gov/science-research/real-world-evidence/real-world-evidence-submissions-center-drug-evaluation-and-research

[31] FDA. Submitting Documents Using Real-World Data and Real-World Evidence to the Food and Drug Administration for Drugs and Biologics [Internet]. 2019 [cited 2024 May 23]. Available from: https://www.regulations.gov/docket/FDA-2019-D-1263/document

[32] FDA. Data Standards for Drug and Biological Product Submissions Containing Real-World Data [Internet]. Federal Register. 2024 [cited 2024 May 23]. Available from: https://www.federalregister.gov/documents/2023/12/22/2023-28291/data-standards-for-drug-and-biological-product-submissions-containing-real-world-data-guidance-for

[33] FDA. Submitting Documents Using Real-World Data and Real-World Evidence to FDA for Drug and Biological Products [Internet]. FDA; 2022 [cited 2025 Mar 21]. Available from: https://www.fda.gov/regulatory-information/search-fda-guidance-documents/submitting-documents-using-real-world-data-and-real-world-evidence-fda-drug-and-biological-products

