# Supplementary material for: Real-World Evidence in FDA Approvals for Labeling Expansion of Small Molecules and Biologics
Source: Ther Innov Regul Sci. 2025 Jun 4;59(5):982–92. doi: 10.1007/s43441-025-00816-9 (PMC12446098; doi:10.1007/s43441-025-00816-9)
Supplement: Supplementary file 1 — Supplementary Material 1 [file 43441_2025_816_MOESM1_ESM.docx]

**Supplemental table 1.** Standardized data abstraction items.

| **Data Item** | **Description** |
| --- | --- |
| **Basic information of product** | Drug name, active ingredients, product types, approval data, review priority, designation, therapeutic area, original indication, original indicated population, pivotal studies. |
| **Labeling change** | Purpose of labeling change, and its contents. |
| **RWE characteristics** | Objective of RWE   - Support safety and/or efficacy assessment   Origin of RWE   - Whether RWE appeared in label and/or FDA review documents - Number of RWE   Characteristics of RWE   - Study temporality: retrospective, prospective, or hybrid - Study design: Case series, Cohort study, External control, Natural history study - Data sources: electronic health record (EHR), Claims, registry, and/or Patient-generated health data (PGHD)   Quality of RWE   - Methods of bias assessment (if reported) - Methods for handling missing data - Comments from FDA reviewers (if available) |

| **Supplemental table 2.** Characteristics of *FDA supplemental approvals for labeling expansion*, 2022-2024 (N=218) | | |
| --- | --- | --- |
| **Characteristic** | **Approvals with RWE** | **Total approvals for labeling expansion** |
|  | *(n=55)* | *(n=218)* |
|  | *n (%)*^a^ | *n* |
| Purpose of approval |  |  |
| Add an indication | 43 (30.28) | 142 |
| Expand the intended population | 12 (15.79) | 76 |
| Product type |  |  |
| NDA | 38 (26.95) | 141 |
| BLA | 17 (22.08) | 77 |
| Approved year |  |  |
| 2022 | 20 (23.26) | 86 |
| 2023 | 26 (27.66) | 94 |
| 2024 | 9 (23.68) | 38 |
| Designation^b^ |  |  |
| Orphan drug | 17 (24.29) | 70 |
| Pediatric | 7 (21.21) | 33 |
| Genetic | 7 (28.00) | 25 |
| \| Abbreviation: RWE, Real-world evidence; FDA, U.S. Food and Drug Administration; NDA, new drug application; BLA, biologics license application \| \| --- \| \| ^a^ All percentages were calculated using the total approvals for labeling expansion in each row.  ^b^ Each designation was not mutually exclusive, one supplemental approval could have more than one designation. \| | | |


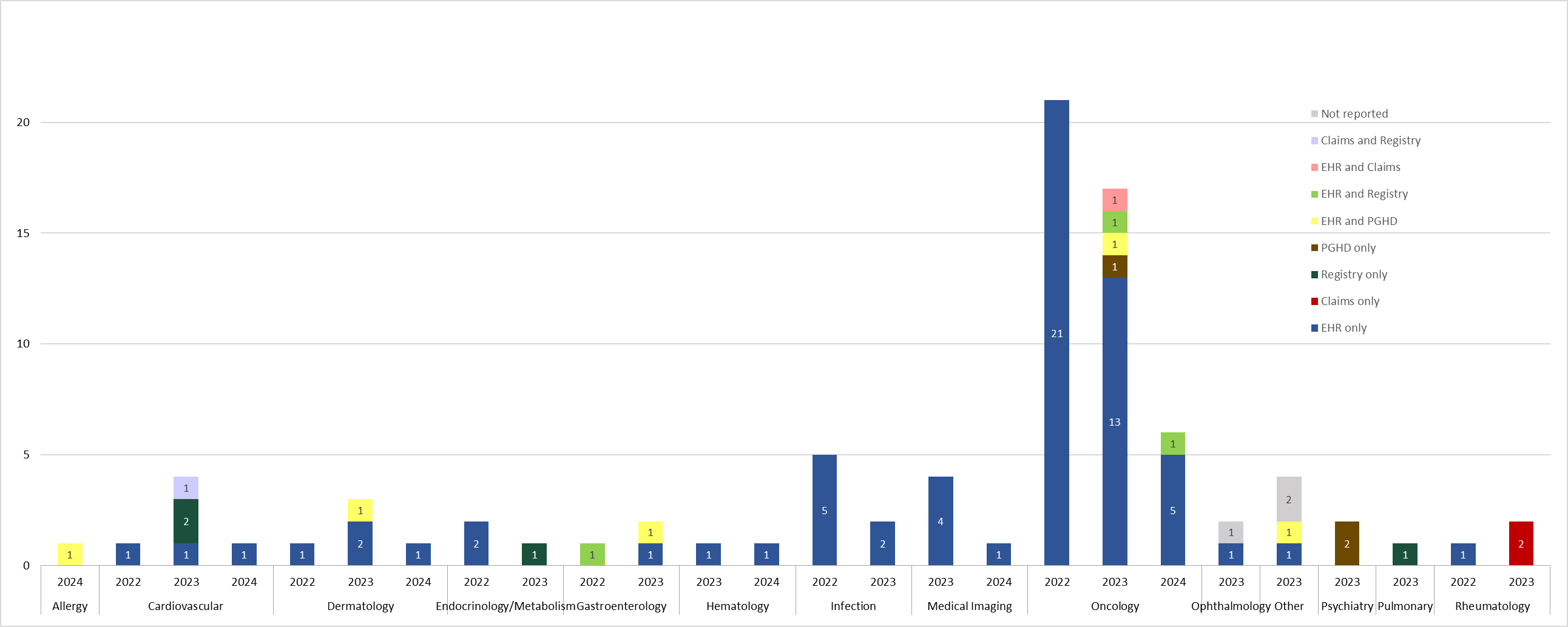


**Supplemental figure 1.** Data Sources for RWE by Year and Therapeutic Area. Abbreviation: EHR: Electronic Health Record; PGHD: Patient-generated Health Data.
